# Optimizing the Quality of Clinical Data in an Australian Aged Care and Disability Service to Improve Care Delivery and Clinical Outcomes: Protocol for an Agile Lean Six Sigma Study

**DOI:** 10.2196/39967

**Published:** 2023-03-27

**Authors:** Lakkhina Troeung, Gap Tshering, Rebecca Walton, Angelita Martini, Martin Roberts

**Affiliations:** 1 Brightwater Research Centre Brightwater Care Group Inglewood Australia; 2 Technology Services Brightwater Care Group Inglewood Australia

**Keywords:** aged care, disability, information technology, data, quality, health services

## Abstract

**Background:**

In Australia, aged care and disability service providers are legally required to maintain comprehensive and accurate clinical documentation to meet regulatory and funding requirements and support safe and high-quality care provision. However, evidence suggests that poor-quality clinical data and documentation are widespread across the sector and can substantially affect clinical decision-making and care delivery and increase business costs.

**Objective:**

In the Optimizing the Quality of Clinical Data in an Australian Aged Care and Disability Service to Improve Care Delivery and Clinical Outcomes (OPTIMISE) study, we aim to use an Agile *Lean Six Sigma* framework to identify opportunities for the optimization of clinical documentation processes and clinical information systems, implement and test optimization solutions, and evaluate postoptimization outcomes in a large postacute community-based health service providing aged care and disability services in Western Australia.

**Methods:**

A 3-stage prospective optimization study will be conducted. Stage 1 (baseline [T_0_]) will measure existing clinical data quality, identify root causes of data quality issues across services, and generate optimization solutions. Stage 2 (optimization) will implement and test changes to clinical documentation processes and information systems using incremental Agile sprints. Stage 3 (evaluation) will evaluate changes in primary and secondary outcomes from T_0_ to 12 months after optimization. The primary outcome is the data quality measured in terms of defects per unit, defects per million opportunities, and Sigma level. The secondary outcomes are care delivery (direct care time), clinical incidents, business outcomes (cost of quality and workforce productivity), and user satisfaction. Case studies will be analyzed to understand the impact of optimization on clinical outcomes and business processes.

**Results:**

As of June 1, 2022, stage 1 commenced with T_0_ data quality audits conducted to measure current data quality. T_0_ data quality audits will be followed by user consultations to identify root causes of data quality issues. Optimization solutions will be developed by May 2023 to inform optimization (stage 2) and evaluation (stage 3). Results are expected to be published in June 2023.

**Conclusions:**

The study findings will be of interest to individuals and organizations in the health care sector seeking novel solutions to improve the quality of clinical data, support high-quality care delivery, and reduce business costs.

**International Registered Report Identifier (IRRID):**

DERR1-10.2196/39967

## Introduction

### Background

In Australia, aged care and disability service providers are legally required to maintain comprehensive and accurate documentation of the care provided to each client [1,2]. This clinical documentation is crucial to support safe and high-quality care delivery, maintain professional accreditation, and acquire government funding [3-5]. However, widespread clinical documentation and data quality issues have been identified across the sector, with evidence suggesting that most existing clinical data in the aged care and disability services sector are of substandard quality and lack consistency within and across individual organizations [3,6,7].

This problem has been partly because of the absence of national data standards and minimum requirements to guide information collection and documentation processes [3,7]. Therefore, service providers are challenged to collect extensive clinical information to meet multiple funding and regulatory reporting requirements across different and often segregated information systems [3,7]. Existing clinical information systems have also largely used “shrink-wrapped” or off-the-shelf systems owned by external vendors that are modeled on designs from other health care environments, with limited customization to account for the specific environment, workflows, and information requirements of aged care and disability services [8-10].

Investigations of workflow in Australian residential aged care facilities (RACFs) have shown that clinical documentation and information processing are major time-intensive staff activities [9,11]. A study involving 6 RACF sites in New South Wales and Victoria estimated that registered nurses spent a median of 60 minutes on documentation per shift, whereas service managers spent a median of 360 minutes on documentation activities, including filling forms, progress notes, incident reporting, and medication management [9]. Similarly, a second study estimated that RACF support workers spent a mean 14.5% of the total working time per shift on documentation tasks, which is equivalent to approximately 60 minutes for a standard 7.5-hour shift [11].

Although documentation is an essential activity of health care provision, excess time spent on documentation directly reduces the amount of care time spent with residents, which can compromise the quality of care delivery [12]. A survey in the United Kingdom found that 81% of the nurses believed they spent a disproportionate amount of time on record keeping and documentation tasks, which prevented them from providing direct care [13]. Other research has shown that the clinical documentation burden is a major driver of burnout among clinical and care staff [14,15], who believe that time could be better spent attending to residents [16-18], and can negatively impact job satisfaction [17].

Health IT (HIT) has a great capacity to support efficient information processing in aged care and disability services and facilitate high-quality person-centered clinical decision-making at the point of care [19,20]. Research has shown that the overall workforce and management perception of the use of electronic health records (EHRs) in aged care is positive, with widespread agreement that EHRs are beneficial for improving workforce efficiency [21]. However, there is a need to optimize HIT systems based on a detailed understanding of the workflow and requirements specific to aged care and disability services [9]. Several pre-post implementation studies in Australian RACFs have shown that the implementation of electronic systems alone does not automatically lead to greater efficiency [11,12,19] or data quality improvement [22] compared with paper-based systems. Instead, the implementation of suboptimal HIT systems can lead to unintended adverse consequences, including increased documentation time, difficulty in data entry and information retrieval, increased complexity of information management, and increased documentation burden and business costs [23].

Several common issues with EHR systems used in aged care and disability services have been described in the literature. End users have reported that existing systems lack clarity and contain inconsistent data fields, definitions, and terminology that do not match the specific information requirements for aged care and disability services [3,19]. When relevant input fields are unavailable, information is either omitted or recorded elsewhere, meaning that the EHR is often incomplete and does not contain all essential information necessary for the delivery of safe care [24]. Systems have also been reported to be difficult to navigate [25] and lack the structure and organization to guide the workforce to record the required clinical information efficiently [7,20]. A clinical documentation audit study in 7 Australian RACFs showed that although EHRs contained a greater *quantity* of information than paper-based records, information recorded in EHRs had a lower total mean *quality* score [22].

Other research has shown that end users can become reluctant to use EHR systems if they do not easily integrate into their workflow and often revert to using paper-based documentation [3]. This creates further inefficiency with respect to double documentation and the duplication of effort, contributing to a “vicious cycle” [19] of increased documentation burden and poorer data quality [3,9]. One study found that RACF nurses reported an excessive amount of time spent entering the same information across multiple incompatible systems and that double documentation resulted in the omission of important information, inaccuracy, and potential safety concerns [24].

In addition to EHR system–related issues, other complex process-related issues have been described that can contribute to missing, incomplete, inaccurate, or not up-to-date clinical data. In the primary care setting, for example, workload and time constraints [26], workforce attitudes toward documentation tasks, and prioritization of direct care tasks [27] have all been shown to affect the quality of nursing documentation. Well-designed EHR systems can support higher-quality clinical data by guiding the workforce to input the required information more efficiently but may not address all process-related barriers related to clinical documentation.

Ultimately, as organizations become more data mature, important business decisions are increasingly reliant on analytics using administrative, clinical, and other service data [28]. Poor data quality can result in flawed business decisions and increased business costs [29]. Therefore, there is a clear need to optimize clinical documentation processes and clinical information systems to support efficient, safe, and quality care delivery and effective business decision-making in aged care and disability services [9].

However, any form of organizational change in systems or processes represents a major cost for service providers, which can be a major barrier to implementation. Therefore, it is important to be able to clearly demonstrate the added value and cost benefits of transformation initiatives to organizational leadership [19]. Currently, few aged care and disability service providers collect benchmarks to routinely evaluate and monitor data quality or its impact on care delivery and organizational efficiency [30]. Implementation without routine evaluation or a control plan can substantially increase business costs, while adding little value to care delivery or operational efficiency.

### The Optimizing the Quality of Clinical Data in an Australian Aged Care and Disability Service to Improve Care Delivery and Clinical Outcomes Study

The overarching goal of the Optimizing the Quality of Clinical Data in an Australian Aged Care and Disability Service to Improve Care Delivery and Clinical Outcomes (OPTIMISE) study is to use an integrated Agile *Lean Six Sigma* (LSS) framework [31,32] to (1) identify opportunities for the optimization of clinical documentation processes and clinical information systems, (2) implement and test optimization solutions, and (3) evaluate postoptimization outcomes in a large postacute community-based health service providing aged care and disability services in Western Australia.

### Objectives

Specifically, the 3-stage study will include the following:

1. Stage 1 (*baseline*): measure existing clinical data quality, identify the root causes of data quality issues, and generate optimization solutions
2. Stage 2 (*optimization*): implement and test changes to clinical information systems and clinical documentation processes
3. Stage 3 (*evaluation*): evaluate changes in clinical data quality, care delivery, clinical outcomes, and business costs following optimization

Table 1 presents the specific study objectives mapped to the Agile LSS phases (see the *Study Design and Framework section*).

**Table 1:** Objectives of the Optimizing the Quality of Clinical Data in an Australian Aged Care and Disability Service to Improve Care Delivery and Clinical Outcomes study.

Stage and phase		Objective
**Stage 1: baseline**		
	1.1. Define	Define study goals, scope, outputs, and methodology
	1.2. Measure	Measure clinical data quality across aged care and disability services
	1.3. Analyze	Identify root causes of data quality issues
	1.4. Improve (part 1)	Generate solutions to support optimization of clinical documentation processes and clinical information systems
**Stage 2: optimization**		
	2.1. Improve (part 2)	Implement and test changes to clinical documentation processes and clinical information systems specific for aged care and disability services
	2.2. Control	Establish policies and procedures for clinical data governance, collection, and input across care services
	2.2. Control	Establish a control plan for routine audit of clinical data quality across care services
**Stage 3: evaluation**		
	3.1. Evaluation	Measure change in primary outcomes (clinical data quality)
	3.1. Evaluation	Measure change in secondary outcomes (care delivery, clinical outcomes, business costs, and workforce satisfaction)

## Methods

### Study Design and Framework

The OPTIMISE study will be a prospective optimization study using an integrated Agile LSS Define, Measure, Analyze, Improve, Control (DMAIC) framework.

*Six Sigma* is a statistical measurement-based method for process optimization and quality improvement that aims to reduce the number of *defects* in a process to <3.4 defects per 1 million opportunities [33]. *Lean* methodology is focused on reducing *waste* in a process, which is defined as any unnecessary or suboptimal items, actions, tasks, components, materials, systems, or human resources that increase costs and time spent on a process [34]. Therefore, the *Lean Six Sigma* framework aims to reduce both *defects* and *waste* to improve the quality of products and services, improve efficiency, and reduce business costs [35,36]. Originally conceptualized in the manufacturing industry, LSS has been increasingly applied in health care to control rising costs and improve the quality of care delivery [30,36-38].

The strength of LSS is its rigorously structured framework for quality improvement based on statistical measurement. However, this structure can lack flexibility to accommodate changes across the project cycle [32]. *Agile* methodology can complement LSS to increase flexibility and adaptability [39]. Agile is a popular methodology used in software development that involves the iterative development of a product over short-term incremental cycles called sprints [31]. It is a highly flexible and responsive methodology that allows for product changes during development based on customer feedback. However, this method neglects decision-making based on objective and measurable data, which can compromise quality and efficiency. Therefore, the integration of Agile and LSS frameworks provides a structured methodology for product development, evaluation, and improvement that is simultaneously flexible and responsive to changes over iterative cycles. Hybrid Agile LSS frameworks have been increasingly used in the literature as a more powerful method for quality improvement than single frameworks [39-42].

### Study Setting

The study will be undertaken at Brightwater Care Group (“Brightwater”), a large postacute, residential, and community-based aged care and disability service in Perth, Western Australia. Brightwater provides residential and home-based care for approximately 1882 aged care and 522 disability services clients across 10 different programs (Table 2).

**Table 2:** Overview of aged care and disability services.

Service type and program name		Sites, n	Clients, n	Description
**Aged care**				
	RAC^a^	11	750	Long-term or permanent high care accommodation for people aged >65 years
	SDCP^b^	1	31	Long-term or permanent high care accommodation for people with dementia
	TCP^c^	2	101	Short-term, posthospital support and active management for older people aged >65 years
	AH^d^	—^e^	1000	Home-based support for people aged >65 years
**Disability**				
	TRP^f^	1	53	Specialist neurorehabilitation service for people aged 18-65 years with acquired brain injury
	TAP^g^	1	23	Short-term, posthospital support and active management for people aged 18-65 years with disability
	SIL^h^	8	71	Long-term and permanent high care accommodation for people aged 18-65 years with disability
	CAPB^i^	—	375	Home-based support for people aged 18-65 years with disability and NDIS^j^ funding

^a^RAC: residential aged care.

^b^SDCP: Specialist Dementia Care Program.

^c^TCP: Transitional Care Program.

^d^AH: at home.

^e^AH and CAPB services are at-home and community-based care sites.

^f^TRP: Transitional Rehabilitation Program.

^g^TAP: Transitional Accommodation Program.

^h^SIL: Supported Independent Living.

^i^CAPB: capacity building.

^j^NDIS: National Disability Insurance Scheme.

### Patient and Public Involvement

Patients or the public were not involved in the design, conduct, reporting, or dissemination plans of our research, as the end product (clinical information system) is not directly used by patients or the public.

### Ethics Approval

The study protocol was approved by Brightwater Care Group as a Level 2: Low Risk Study (reference: 2021/BCG2110). This study is classified as an internal service evaluation and does not require ethics approval for research in Australia.

### Consent and Participation

Client EHR data will be deidentified before the analysis. Clients provided written consent for their deidentified clinical data to be used for research and service evaluation as part of the conditions of entry into the service. Staff member participation in user surveys and interviews will be voluntary, and nonparticipation will not impact their usual role. All participant data will be deidentified by assigning a randomized numbered code. Participant names will be changed to pseudonyms when referring to qualitative data. When transcribing interviews verbatim, words or statements that could be used to identify participants will be removed to ensure privacy. Audio recordings will be deleted once the transcriptions are complete.

### Study Phases

#### Overview

**Figure 1:**
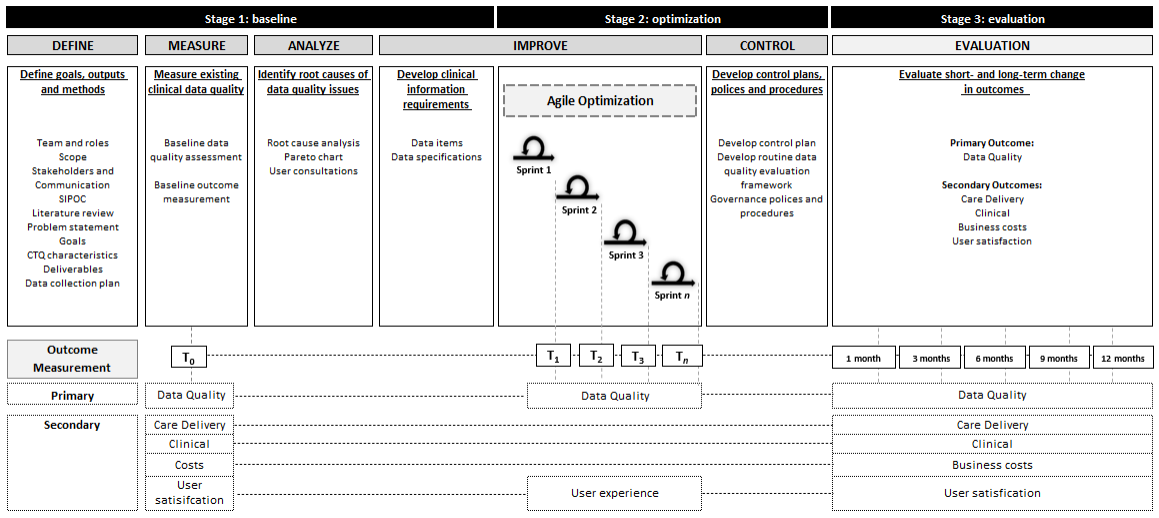
The Optimizing the Quality of Clinical Data in an Australian Aged Care and Disability Service to Improve Care Delivery and Clinical Outcomes study design. CTQ: critical-to-quality; SIPOC: Suppliers, inputs, process, outputs, customers.

Figure 1 outlines DMAIC phases of the study. Stage 1 (*baseline*) will measure existing clinical data quality across services, identify root causes of data quality issues, and generate solutions to directly inform optimization. Stage 2 (*optimization*) will implement and test changes to clinical documentation processes and information systems specific to aged care and disability services. Stage 3 (*evaluation*) will measure the short-term (1-3 months) and long-term (4-12 months) changes in data quality and clinical and business outcomes following optimization.

#### Stage 1: Baseline

##### Phase 1.1: Define

To execute the study, a multidisciplinary *Six Sigma* project team [43] was formed, consisting of 5 *regular* team members—a project champion (director of research), process owner and expert (technology services manager), project leader (data scientist), project manager and analyst (information systems analyst), and process user (clinical expert). The project leader has >8 years of experience in leading evaluation projects using complex clinical data including hospital, emergency department, general practice, pharmaceutical, Medicare, mortality, aged care, and disability data, whereas the project manager and analyst holds >6 years of experience in project management, policy analysis, and information systems analysis, and both have Black Belt certifications in LSS. The process user is a research-trained occupational therapist with 3 years of experience providing clinical care for residents at 1 of the residential aged care sites involved in the study and was included as a regular team member to provide clinical oversight and expertise as a direct user of clinical information systems.

In addition, 5 executive and senior managers were included as *resource* and ad hoc team members to provide high-level information and process expertise, as needed.

Several different LSS techniques including Voice of Customer; 5 Whys; Specific, Measurable, Achievable, Realistic, and Timely goals; stakeholder analysis; and a Suppliers, Inputs, Process, Outputs, and Customer diagram were used to define study goals, scope, outputs, critical-to-quality (CTQ) characteristics, and methodology [43]. In addition, a Failure Modes and Effects Analysis [44] was conducted to identify the potential risks and failures. Multimedia Appendix 1 provides a brief description of the LSS techniques that appear in this manuscript for readers who are unfamiliar with the LSS framework.

The main component of the define phase was to define the CTQ metrics to be used to measure data quality in the study. CTQ metrics are key factors or attributes determined to be important by an organization and are used to measure the performance of a process [45]. The CTQ metrics for the clinical data were determined using the Delphi consensus method [46]. First, a literature review was conducted to identify data quality metrics commonly used in data quality assessments [47-49]. After review by the project team, a shortlist of the 15 most relevant metrics was circulated to an expert group consisting of 68 key stakeholders in the organization across executives, care services, clinical excellence, quality, technology, business analytics, and research departments, who independently ranked metrics in order of those most critical to clinical data quality in their role. The final CTQ metrics represented the 6 metrics with the highest rankings among the expert group (Table 3).

The define phase was completed in January 2022. The subsequent sections outline the planned protocol for the remaining phases.

**Table 3:** Critical-to-quality metrics used to measure clinical data quality.

Rank	Metric	Definition of metric	Measurement method	Defect definition
1	Accuracy	The degree to which EHR^a^ data correctly represent a client’s personal, medical, clinical, and psychosocial circumstances and care needs	Manual audit of random sample of EHRs compared with original documents (eg, admission documents or medical reports)	Data that do not match the original source of truth
2	Completeness	The degree to which all required data in the EHR are present	Data warehouse audit of all EHRs	Missing data (ie, null or blank fields)
4	Currentness	The degree to which EHR data are up to date and reflect the client’s current condition and changes in circumstances and care needs	Data warehouse audit of all EHRs	Data fields not updated within required timeframes as per clinical guidelines
3	Clarity	The degree to which data are presented in a clear format and enable the user to understand a client’s care needs without ambiguity	Manual assessment of a random sample of EHRs	Data fields with unclear presentation
5	Compliance	The degree to which EHRs capture all the required information to meet legal, funding, and regulatory requirements and in accordance with best practice clinical guidelines	Manual review of data fields captured in existing systems	Missing mandatory data (ie, null or blank fields)
6	Usability	The degree to which data are presented in a format that allows the information to be directly and efficiently used for primary (eg, care provision) and secondary purposes (eg, reporting, analytics, and evaluation)	Data warehouse audit of all EHRs	Data with limited primary and secondary usability

^a^EHR: electronic health record.

##### Phase 1.2: Measure

The measure phase will focus on measuring the baseline (T_0_) quality of clinical data across services and identifying key data quality issues. T_0_ data quality will be measured through an audit of client EHRs using a 6-month lookback period. Data quality will be measured in terms of the *number of defects* present in EHRs, measured using the CTQ metrics identified by the expert group.

Specifically, T_0_ data quality will be measured by the number of defects per unit (DPU), defects per opportunity (DPO), defects per million opportunities (DPMO), and the Sigma level (Table 4). T_0_ data quality will be measured for each of the 8 programs to allow the identification of any systematic variation between sites and service-specific data quality issues. In addition, the assessment will identify the most common types of defects across services to inform system optimization.

**Table 4:** *Six Sigma* metrics for measuring defects.

Metric	Formula	Description
DPU^a^	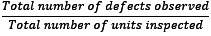	Measures the average number of defects present in a unit (ie, the average number of defects in each client EHR^b^)
DPO^c^	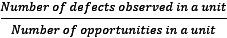	Measures the number of defects as a proportion of the total number of data fields (ie, opportunities) present in each client EHR
DPMO^d^	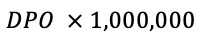	Measures the number of DPO expressed per million
Sigma level	Determined from conversion tables using calculated DPMO	Measures the amount of variability in a process. *Six Sigma* quality performance is defined as 3.4 DPMO.

^a^DPU: defects per unit.

^b^EHR: electronic health record.

^c^DPO: defects per opportunity.

^d^DPMO: defects per million opportunities.

##### Phase 1.3: Analyze

Following the identification of key data quality issues across services, the analyze phase will focus on understanding the *root causes* of data quality issues. A qualitative research approach will be used to understand the workforce experiences of clinical documentation processes using existing clinical information systems and to identify the root causes of poor data quality.

A purposive sample of the workforce across all 8 programs will be selected to participate in user consultations using either semistructured interviews or web-based surveys (Multimedia Appendix 2). Different functional user groups will be purposively sampled at each site (care staff and clinical, administrative, and service managers) to provide whole-of-organization insight into existing processes, strengths, and gaps. At least 1 site from each program will be selected (a minimum of 8 sites). For programs with multiple sites, the sites with the highest, median, and lowest data quality based on the findings mentioned in *Phase 1.2: Measure* will be selected for consultation. In addition, users from the corporate workforce who routinely use clinical data or clinical information systems for reporting and analytics will also be consulted to provide insight into the back-end quality of clinical data.

Data collected through user consultations will be used to generate initial subthemes and themes using an inductive thematic analysis approach [50]. Generated themes and subthemes will then be assembled into a fishbone diagram [51], which is an LSS visualization technique to identify the root causes of poor data quality across services.

##### Phase 1.4: Improve (Part 1)

In the final phase of stage 1, information collected in the measure and analyze phases will be used to generate solutions to optimize clinical data quality across services. Solutions will address identified process-related issues (eg, deprioritization of documentation tasks and workforce roles and responsibilities) and system-related issues (ie, system design, performance, and usability).

Program-specific *data requirements* and *priorities* will be established to meet funding and regulatory requirements, best practice clinical guidelines, and the specific workflows of each service. In addition, data specifications will be developed to enable efficient and accurate information capture to enable high-quality primary (eg, care delivery) and secondary (eg, reporting, research, and evaluation) uses of data. A solutions selection matrix [43] will be used to propose and rank solutions according to perceived cost benefits and to prioritize recommendations for implementation based on the most urgent needs of the organization.

#### Stage 2: Optimization

##### Phase 2.1: Improve (Part 2)

Stage 2 will use Agile methodology to test and implement changes to clinical documentation processes and clinical information systems over a 12-month period.

Program-specific data optimization will be conducted to meet the specific workflows and funding, regulatory, and clinical requirements of each service. *Data requirements* and *priorities* established in stage 1 will determine the order of the Agile sprints (ie, incremental cycles of changes). At the end of each sprint, data quality will be evaluated by calculating DPU, DPO, DPMO, and the Sigma level statistics as mentioned in the *Phase 1.2: Measure* section. User acceptance, experience, and satisfaction will be evaluated through user surveys, and user feedback from each sprint will be directly incorporated into the next Agile sprint. This iterative optimization method ensures a flexible approach that incorporates both user feedback and statistical measurement.

##### Phase 2.2: Control

At the end of stage 2, governance policies and procedures and a control plan will be developed to allow the process owner to routinely monitor and evaluate the quality of clinical data across the service after implementation.

The control phase is the final phase and a critical part of the LSS DMAIC framework, which ensures that a new or improved process continues to work successfully after its implementation as a regular business process. A *Clinical Data Quality Audit Tool* will be designed to allow the routine evaluation of data quality across services as part of ongoing business practice. Internal policy and procedure documents will be developed, and training will be provided to the relevant departments.

#### Stage 3: Evaluation

The final stage of the study will evaluate changes in the primary and secondary outcomes in the 12 months following the full optimization (Table 5). Outcomes will be measured at T_0_ and at 1, 3, 6, 9, and 12 months after full optimization. Previous studies in Australian RACFs have shown that the time spent on documentation tasks generally increases in the period after the implementation of new or changed systems as users learn the new technology and adjust their workflows [11,12]. Therefore, long-term evaluation is critical for reliably quantifying changes in outcomes and their impact on organizational processes.

**Table 5:** Primary and secondary outcomes and data collection plan.

Outcomes and data source or method					Time point																
					Baseline (T_0_)		Sprints 1 to n						Follow-up								
							T_1_		T_2_		T_n_		T_1 month_		T_3 months_		T_6 months_		T_9 months_		T_12 months_
**Primary outcome**																					
	**Data quality**																				
		**DPU^a^**																			
			EHR^b^ audit	✓		✓		✓		✓		✓		✓		✓		✓		✓	
		**DPO^c^**																			
			EHR audit	✓		✓		✓		✓		✓		✓		✓		✓		✓	
		**DPMO^d^**																			
			EHR audit	✓		✓		✓		✓		✓		✓		✓		✓		✓	
		**Sigma level**																			
			EHR audit	✓		✓		✓		✓		✓		✓		✓		✓		✓	
**Secondary outcomes**																					
	**Care delivery**																				
		**Direct care time**																			
			Self-report survey	✓								✓		✓		✓		✓		✓	
		**Documentation time**																			
			Self-report survey	✓								✓		✓		✓		✓		✓	
		**Other activities**																			
			Self-report survey	✓								✓		✓		✓		✓		✓	
	**Clinical outcomes**																				
		**Pressure injury^e^**																			
			EHR audit	✓								✓		✓		✓		✓		✓	
		**Restraints^e^**																			
			EHR audit	✓								✓		✓		✓		✓		✓	
		**Falls^e^**																			
			EHR audit	✓								✓		✓		✓		✓		✓	
		**Weight loss^e^**																			
			EHR audit	✓								✓		✓		✓		✓		✓	
		**Medication management^e^**																			
			EHR audit	✓								✓		✓		✓		✓		✓	
		**Behaviors of concern**																			
			EHR audit	✓								✓		✓		✓		✓		✓	
		**Choking**																			
			EHR audit	✓								✓		✓		✓		✓		✓	
		**Infection**																			
			EHR audit	✓								✓		✓		✓		✓		✓	
		**Unplanned hospital admission**																			
			EHR audit	✓								✓		✓		✓		✓		✓	
		**Wounds**																			
			EHR audit	✓								✓		✓		✓		✓		✓	
	**Business outcomes**																				
		**Cost of quality**																			
			Cost analysis	✓																✓	
		**Workforce productivity**																			
			Self-report survey	✓								✓		✓		✓		✓		✓	
	**End user**																				
		**User satisfaction**																			
			Self-report survey	✓								✓				✓				✓	
		**User experience**																			
			Self-report survey and interviews			✓		✓		✓										✓	

^a^DPU: defects per unit.

^b^EHR: electronic health record.

^c^DPO: defects per opportunity.

^d^DPMO: defects per million opportunities.

^e^National Quality Indicator Program (NQIP) outcome.

### Primary Outcome

The primary study outcome is data quality measured using DPU, DPO, DPMO and Sigma level. Improvement in data quality from T_0_ to 12 months after optimization will be determined by a reduction in DPU, DPO, and DPMO and an increase in Sigma level. A well-performing process should operate at a Sigma level of 6 and have ≤3.4 DPMO [43].

### Secondary Outcomes

#### Care Delivery

Care delivery will be measured by the proportion of weekly direct care time spent with residents using a self-report version of the Work Measurement Tool developed by Munyisia et al [52], which measures 8 categories of care activities in Australian RACFs, including *direct care, medication management, communication, documentation, indirect care, personal, in-transit*, and *other*. Clinical and care staff will be invited to complete self-report surveys from T_0_ to 12 months after optimization to provide a measure of workflow before and after optimization. Self-reported activities will be validated against observer-rated activities for a random staff subsample.

#### Clinical Outcomes

Under the National Quality Indicator Program, Australian aged care providers are required to undertake mandatory reporting of 5 National Quality Indicator Program indicators as a measure of the quality of clinical services and care provision [53]. These include *pressure injuries, physical restraints, falls, unplanned weight loss*, and *medication management*. In addition, Brightwater routinely measures 5 internal clinical indicators of quality across both aged care and disability services (behaviors of concern, choking, infection, unplanned hospital admission, wounds; Table 5). This study will measure changes in clinical outcomes from T_0_ to 12 months after optimization.

#### Business Outcomes

A cost of quality (CoQ) analysis [54] will be undertaken to compare the operational costs before and after optimization. The CoQ analysis is a critical part of the LSS framework to estimate the ongoing operational costs of optimizing the data or information system and to ensure that the expenses associated with achieving higher data quality are balanced against the costs of poor quality, which is defined as expenses incurred on resources and non–value-added activities to fix poor-quality data. CoQ costs include costs incurred on the optimization, ongoing operation, and maintenance of the data or information system, whereas poor-quality costs include costs incurred on activities such as data cleaning, data quality inspection, and meetings to rectify data inconsistencies. A health economist will be employed to undertake the CoQ analysis.

In addition, workforce productivity will be evaluated before and after optimization in relation to regulatory reporting. Corporate staff will be invited to complete self-report surveys from T_0_ to 12 months after optimization to provide an estimate of the number of hours worked to complete regulatory reporting activities before and after optimization.

#### User Satisfaction

Finally, self-report surveys and qualitative interviews will be used to measure changes in user satisfaction with clinical data systems before and after optimization. Prior research has shown that clinical documentation burden is a major driver of burnout among care staff [14,15] and can negatively impact job satisfaction [17]. Therefore, user satisfaction is an important outcome of this study.

### Quantitative Analysis

Quantitative analyses will be conducted using STATA (version 16.0; StataCorp LLC) [55]. Multilevel mixed effects regression models will be used to evaluate any change in primary and secondary outcomes from T_0_ to 12 months after optimization. For clustered longitudinal data, multilevel modeling recognizes that a change in outcome is affected by a fixed effect (ie, implemented changes) and random effects at both individual and group levels and can explicitly account for this multilevel random variation [56].

An a priori power calculation was performed to determine the minimum number of EHRs required to be evaluated to detect a significant change in our primary outcome (ie, data quality measured using DPU, DPO, DPMO, and Sigma level) from T_0_ to 12 months after optimization. First, we used G*Power [57] to compute the required sample size to detect a medium difference (*f*=0.15) at a Cronbach α level of .05 and a power level of .80 using a linear multiple regression analysis with *k*=3 fixed predictors (time, program, and the time × program interaction), which returned a required sample size of 77.

This was multiplied by the anticipated *design effect* [58], which is an adjustment factor for clustering in multilevel models. The design effect is calculated as:

1 + [(*n −* 1) × ICC]                       **(1)**

where n is the expected number of subjects per cluster and “ICC” is the intracluster coefficient or the expected correlation within clusters [58]. We defined a cluster as a facility (ie, site) with a median cluster size of 30 clients per facility. With an anticipated moderate intracluster coefficient of 0.10, the design effect is equal to 3.9, giving a required sample size of 77 × 3.9 = 300. On the basis of the occupancy rates and client population size (n=2200) as of January 2022, our primary outcome analysis will have sufficient statistical power.

### Qualitative Analysis

Qualitative data from user interviews and surveys will be coded and analyzed using NVivo 12 (QSR International) [59]. An inductive thematic analysis approach will be used to identify and understand key themes, specifically to classify, order, and reassemble data to identify converging and diverging perspectives. A total of 50 interviews are anticipated to reach saturation.

## Results

As of June 1, 2022, stage 1 commenced with T_0_ data quality audits conducted to identify the current data quality and system strengths and limitations. T_0_ data quality audits will be followed by user consultations to identify root causes of data quality issues. Clinical information requirements will be developed by May 2023 to inform optimization (stage 2) and evaluation (stage 3). Results are expected to be published in June 2023.

## Discussion

It is anticipated that the study findings will show that optimization of clinical data and documentation will, in turn, have a major impact on care delivery and clinical outcomes and reduce business costs. Although the aim of the OPTIMISE study is to build internal organizational capacity for continuous improvement of clinical data quality, the findings will also be important to individuals and organizations across the aged care and disability service sector, as well as the wider health care sector, which is seeking novel technology solutions to improve the quality of clinical data to support high-quality care delivery and operational efficiency. In addition, the findings will be relevant to researchers and organizations interested in learning the opportunities and limitations of engaging an Agile LSS framework for quality improvement in a health care setting. These findings can stimulate organization-level research to support the development of evidence-based care. The findings will also be disseminated nationally and internationally through industry presentations, scientific conference presentations, and peer-reviewed journal publications.

## Appendix

Multimedia Appendix 1 *Lean Six Sigma* techniques.

Multimedia Appendix 2 Interview schedule.

## Abbreviations

CoQ: cost of quality
CTQ: critical-to-quality
DMAIC: Define, Measure, Analyze, Improve, Control
DPMO: defects per million opportunities
DPO: defects per opportunity
DPU: defects per unit
EHR: electronic health record
HIT: health IT
LSS: *Lean Six Sigma*
OPTIMISE: Optimizing the Quality of Clinical Data in an Australian Aged Care and Disability Service to Improve Care Delivery and Clinical Outcomes
RACF: residential aged care facility
T0: baseline

## Data Availability

Data sharing is not applicable for this paper as no data sets were generated or analyzed during this study.
