# Supplementary material for: Optimizing the Quality of Clinical Data in an Australian Aged Care and Disability Service to Improve Care Delivery and Clinical Outcomes: Protocol for an Agile Lean Six Sigma Study
Source: JMIR Res Protoc. 2023 Mar 27;12:e39967. doi: 10.2196/39967 (PMC10132011; doi:10.2196/39967)
Supplement: Multimedia Appendix 1 [file resprot_v12i1e39967_app1.docx]

**Supplementary Table 1. Lean Six Sigma Techniques**

| **LSS Technique** | **Brief Description** |
| --- | --- |
| Voice of Customer | Voice of customer refers to collecting feedback from customers. Feedback may be collected through focus group and interviews, or using electronic methods such as surveys, social media interactions, reviews, etc. |
| 5 Whys | Brainstorming tool that repeats why questions to understand the root cause(s) of a problem. |
| SMART goals | Qualifying goal statement based on indicators that include 1) specific, 2) Measurable, 3) Attainable, 4) Realistic, and 5) Time-bound. |
| Stakeholder analysis | Identification of various individuals, both internal and external to the organisation, who relate to the project or are impacted by the project. |
| Suppliers, Inputs, Process, Outputs, and Customer (SIPOC) diagram | Tool used to understand business processes and interactions between processes. |
| Failure Modes and Effects Analysis (FMEA) | Tool used for assessing and prioritising risk mitigation measure. |
| A Root Cause Analysis | Root cause analysis is the process of identifying main causes of problems. |
| Cause and Effect (Fishbone) Diagram | Fishbone is a specific brainstorming tool for root cause analysis which helps teams to visualise data to identify patterns and relationships. |
| A Root Cause Verification Matrix | Tool used for validating root causes identified through root cause analysis tools. |
| A Solutions Selection Matrix | Analytical tool for ranking validated root causes based on effectiveness, feasibility and cost-benefit. |
