# Supplementary material for: Optimizing the Quality of Clinical Data in an Australian Aged Care and Disability Service to Improve Care Delivery and Clinical Outcomes: Protocol for an Agile Lean Six Sigma Study
Source: JMIR Res Protoc. 2023 Mar 27;12:e39967. doi: 10.2196/39967 (PMC10132011; doi:10.2196/39967)
Supplement: Multimedia Appendix 2 [file resprot_v12i1e39967_app2.pdf]

Interview Schedule

1. **What is your experience of using iCare and/or Carelink?**
2. **We had a chance to go through 10% of client notes and found a number had information that was missing, incorrect, unclear, or not up to date. Why do you think this happens?**
   i. Have you ever looked for certain client information in iCare/Carelink and found that it was <u>missing</u> or wasn't recorded? Why?
   ii. When looking at a client record in iCare/Carelink, how do you know whether the information is up to date? Do you know how regularly/often client information should be updated? What are some reasons why a client's information might not be up to date?
   iii. Have you ever found any incorrect information or had trouble understanding/interpreting any client information in iCare/Carelink? Why?
3. **How easy is it to use client information from iCare/CareLink to support ……………………?**

   Clinical staff: Clinical decision making

   Care staff: Supporting client

   Backend users: Reporting and/or Analytics

   Service managers: Service management decisions and reporting

   Admin: Reporting
4. **What client information is crucial to record to provide person-centred care/reporting/analytics? Makes your job easier? Or not currently collected?**
   i. **Site managers and clinical staff:** reporting and delivering person-centred care?
   ii. **Care staff:** delivering person-centred care?
   iii. **Corporate managers and backend users:** reporting or analytics?
5. **Would you like to share anything else about iCare/CareLink?**
